# Posttraumatic Isolated Right Gluteus Minimus Tear: A Case Report

**DOI:** 10.7759/cureus.23056

**Published:** 2022-03-11

**Authors:** Mohd Yazid Bajuri, Parthiban Sivasamy, G Ruslan N Simanjuntak, Aina Fatini Azemi, M Irfan Azman

**Affiliations:** 1 Department of Orthopaedics & Traumatology, Universiti Kebangsaan Malaysia Medical Centre (UKMMC), Kuala Lumpur, MYS; 2 Department of Orthopaedics, KPJ Healthcare University College, Nilai, MYS; 3 Arthroplasty Unit, Department of Orthopaedics, KPJ Tawakkal Specialist Hospital, Kuala Lumpur, MYS

**Keywords:** gluteus medius, hip joints, greater trochanteric syndrome, isolated tear, gluteus minimus

## Abstract

Greater trochanteric pain syndrome (GTPS) is often diagnosed in patients who present with pain over the lateral aspect of the hip. Trauma with injury to the gluteus minimus and medius muscles results in hip pain, which should be considered when diagnosing chronic pain of the lateral hip. The gluteus minimus tendon insertion is located anterior to the anterior facet of the greater trochanter of the femur anatomically. Hence, gluteus minimus tendon pathology may also manifest as chronic lateral hip pain and is considered as the etiology of GTPS. These conditions do not respond to physiotherapy and analgesia. Both open and keyhole endoscopic methods have produced good results in addressing hip pain and abduction weakness. There is a lack of literature regarding isolated gluteus minimus tendon tear as the cause of chronic lateral hip pain or GTPS. Here, we present a rare case of a middle-aged lady with GTPS due to isolated gluteus minimus injury.

## Introduction

The gluteus minimus and medius muscles are the main abductors of the hip joint, and injury to the tendons connecting these muscles at insertion is increasingly being recognized in patients with lateral hip pain and abduction weakness. This is a scenario observed in elderly women in the fourth to sixth decades of life. The incidence of gluteus minimus tears in women is four times higher than that in men, and previous trauma, osteoarthritis, and obesity are contributing factors for the development of gluteus minimus tears [1,2].

Although the etiology is unclear, a study by William et al. reported that the increased prevalence of up to 25% of late-middle-aged women may be correlated with altered biomechanics due to the differences in the shape, size, and orientation of the pelvis and its relationship with the iliotibial band disorders (ITB) [2] In most patients, an abductor tendon tear is accompanied by trochanteric bursitis, which is often a precursor or a result of gluteus muscle tendinopathy [3]. Thus, trochanteric bursitis is most often the secondary diagnosis in patients with hip gluteus minimus tear.

The most commonly injured hip abductor during trauma is the gluteus medius; however, a recent study by Yanke et al. in 2013 reported that gluteus minimus is also injured when a gluteus medius tear occurs [4]. The possible cause of this tear remains unknown, though chronic degeneration of the muscle-tendon unit is believed to be the cause. If it remains untreated, this degeneration can lead to tendinopathy, preceding the chronic lateral hip pain and, eventually, lead to tearing and retracting from the trochanteric attachment [5]. However, there are no reported cases where the gluteus minimus tendon is torn or injured alone. Steroid injection to the trochanteric bursa or at the peritendinous insertion may provide temporary relief but does not heal tendon tears [4].

## Case presentation

A 42-year-old Malay woman was under the care of our clinic for follow-up for right femoroacetabular impingement with a cam lesion in the femoral head. She had a fall from the stairs three months ago and experienced pain arising from the right lateral aspect of the hip joint with associated right hip weakness. The patient characterized that the pain intensified when standing for a long time, climbing stairs, and standing on the right limb alone, and the worst pain was experienced when she lay in the right lateral position. Before the trauma, the patient had right hip pain with mild locking at the groin associated with external rotation of the right hip but not on the lateral aspect. On physical examination, the patient had severe tenderness upon touching over the greater trochanter; there was tenderness when resisting internal rotation or adduction of the right hip as it increases the tensile load on the gluteus medius and minimus tendons. Moreover, reduced power (4/5) for right hip abduction was observed. However, no Trendelenburg gait or sign was noted.

**Figure 1:**
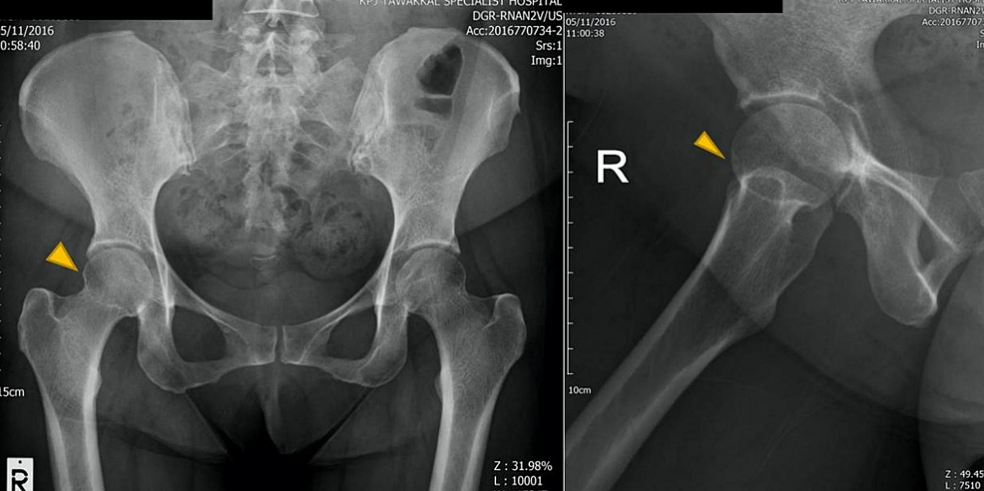
Cam impingement of the right femoral head.

**Figure 2:**
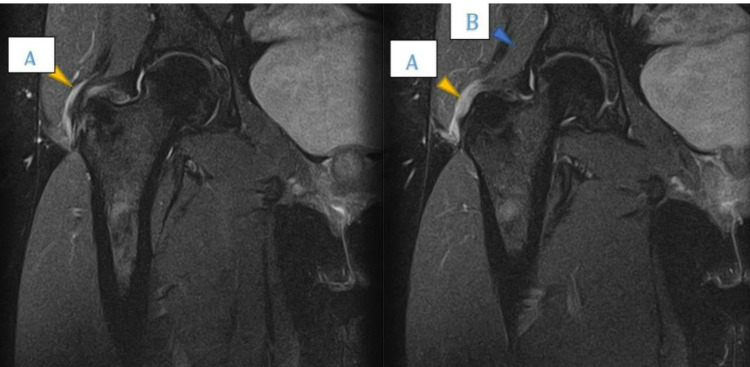
Magnetic resonance images of the right hip (T2-weighted). (A) High-intensity signal at the superolateral aspect of the greater trochanter, showing a partial tear of the gluteus minimus at its insertion. (B) Gluteus minimus muscle.

Radiography showed the preexisting cam lesion in the right femoral head but without evidence of tendon calcification or avulsion fracture at the greater trochanter (Figure 1). Magnetic resonance imaging (MRI) was performed after three months (Figure 2). On T2-weighted images, a high-intensity zone was observed around the greater trochanter, indicating fluid accumulation, and a partial tear of the gluteus minimus at its insertion was observed. There was no significant finding for the gluteus medius muscle and no other injury was noted. The patient received analgesia and specific focus physiotherapy on gluteal strength and control. After three weeks of no symptom improvement, the patient was advised to undergo surgery to repair the gluteus minimus tear.

The patient was placed in the supine position with traction applied to the right lower limb. Surgery was performed under general anesthesia. The right lower limb was cleaned and the hip was draped. The surgery started with diagnostic intraarticular hip arthroscopy. The femoroplasty was performed for the underlying cam lesion of the femoral head. Using the same portal, endoscopes were retracted to visualize the space around the greater trochanter. The trochanteric bursa was resected, providing better visualization, and the torn gluteus minimus was located. The tendon tear orientation suggested that the tendon repair should be performed using the open technique. Through the lateral approach of the hip, a skin incision was made along the greater trochanter and was progressed further to split the iliotibial band longitudinally. The soft tissue was retracted until the lateral part of the greater trochanter was visualized. Then, the assistant maneuvered the traction system to reveal the facet of the anterior trochanter where the gluteus minimus inserts. Using a 5.0-mm endoscopic burr, the tendon insertion footprint was debrided and burred to anchor the 4.5-mm anchor sutures tied to the gluteus minimus tendon. The anchored gluteus minimus tendon was tested for stability using a full arc of motion. Vicryl absorbable sutures were used for fascia and subcutaneous tissue closure. Skin closure was performed with running subcuticular sutures using Monosyn 3/0.

In the ward, the patient was advised to undergo passive physiotherapy, which included hip flexion from 20° to 80° using a continuous passive motion apparatus. Then, the patient was advised to use non-weight-bearing crutches for six weeks and was restricted from performing any active motion of the right hip. During follow-up at six months after surgery, the patient achieved full arc of motion (i.e., internal rotation, 25°; external rotation, 45°; extension, 10°; and flexion, 130°). She regained right hip strength and function, which were comparable to those of the left hip. She was comfortable, and greater trochanteric pain syndrome (GTPS) was relieved.

## Discussion

In the past, most cases of lateral hip pain were misdiagnosed as trochanteric bursitis and conservatively treated with physiotherapy and steroid injections or nonsteroidal anti-inflammatory drugs. While this approach can provide temporary relief, it does not solve the problem in the long term.

Patients often complain of pain at the lateral aspect of the hip, reduced hip abduction strength, and limitations in performing physical activities requiring hip movements, especially in the lateral decubitus position [3]. The presentation is similar to that of trochanteric bursitis; however, considering the reduced hip abduction strength, gluteus minimus or medius tears could be a cause [6].

Recently, the anatomy and characteristic physiology of the peritrochanteric space of the hip has begun to be better understood. The process of diagnosing GTPS can be challenging and complicated as it needs screening of several conditions, such as acute gluteus medius and minimus tears, greater trochanteric bursitis, and snapping hip.

﻿There are various reasons for hip pain. The possibility of diagnosis can be either hip joint-related or not. Ligamentum teres tears, hip labral tears, avascular necrosis of the femoral head, osteophyte loose bodies, cam impingement, pincer lesions, capsular laxity, and cartilage injury are the main causes of hip intraarticular hip pathologies. Extraarticular hip pathologies include neck fractures (hairline or stress), piriformis impingement syndrome, and malignancy [7]. Other conditions contributing to hip pain are superior gluteal neuropathy, lateral femoral cutaneous nerve paresthetica, degenerative lumbar spine, and nerve root impingement. In patients with lumbar spondylosis or lumbar radiculopathy, as in patients with GTPS, antalgic or Trendelenburg gait can be present [8]. Superior gluteal nerve injury could be the cause of hip abductor weakness, especially in patients who have undergone hip surgeries.

MRI is sensitive and accurate in diagnosing tendon tears, which are later confirmed during surgery. Moreover, 27% of tendon tears involve tendon discontinuation [9]. Our patient had a high-intensity signal at the superolateral aspect of the greater trochanter, indicating the discontinuation of the gluteus minimus tendon at its insertion.

Initially, when the diagnosis of gluteal minimus tears is confirmed, commonly practiced conservative treatment modalities can be initiated. When physiotherapy and anti-inflammatory drugs fail to relieve the pain and regain the strength of hip abductors, surgery is indicated; the choice of a surgical approach (i.e., open or arthroscopic approach) is at the orthopedic surgeon’s preference depending on the tear pattern.

## Conclusions

Patients with GTPS usually present with a gradual onset of pain at the lateral aspect of the hip; however, clinicians should consider acute tear or avulsion of the gluteus minimus when dealing with these patients. Gluteus minimus tears are rare and should be treated to relieve hip pain and recover hip activity. To our knowledge, this is possibly the first case of a traumatic isolated gluteus minimus tear reported in the literature.
